# Mortality risk over time after early fluid resuscitation in African children

**DOI:** 10.1186/s13054-019-2619-y

**Published:** 2019-11-27

**Authors:** Elizabeth C. George, Sarah Kiguli, Peter Olupot Olupot, Robert O. Opoka, Charles Engoru, Samuel O. Akech, Richard Nyeko, George Mtove, Ayub Mpoya, Margaret J. Thomason, Jane Crawley, Jennifer A. Evans, Diana M. Gibb, Abdel G. Babiker, Kathryn Maitland, A. Sarah Walker

**Affiliations:** 1 Medical Research Council Clinical Trials Unit (MRC CTU) at UCL, Institute of Clinical Trials and Methodology, UCL, London, UK; 2 Department of Paediatrics, Mulago Hospital, Makerere University, Kampala, Uganda; 3 Mbale Clinical Research Institute, Mbale, Uganda; 4 Kilifi Clinical Trials Facility, KEMRI-Wellcome Trust Research Programme, Kilifi, Kenya; 5 Department of Paediatrics, St Mary’s Hospital, Lacor, Gulu, Uganda; 6 Department of Paediatrics, Joint Malaria Programme, Teule Hospital, Teule, Tanzania; 7 Centre for Tropical Medicine and Global Health, Nuffield Department of Medicine, University of Oxford, Oxford, UK; 8 Department of Paediatrics, University Hospital of Wales, Cardiff, UK; 9 Department of Paediatrics, Faculty of Medicine, Imperial College, Kensington, London, UK

**Keywords:** Mortality risk, Paediatric shock, Fluid resuscitation, Africa, Parametric models

## Abstract

**Background:**

African children hospitalised with severe febrile illness have a high risk of mortality. The Fluid Expansion As Supportive Therapy (FEAST) trial (ISCRTN 69856593) demonstrated increased mortality risk associated with fluid boluses, but the temporal relationship to bolus therapy and underlying mechanism remains unclear.

**Methods:**

In a post hoc retrospective analysis, flexible parametric models were used to compare change in mortality risk post-randomisation in children allocated to bolus therapy with 20–40 ml/kg 5% albumin or 0.9% saline over 1–2 h or no bolus (control, 4 ml/kg/hour maintenance), overall and for different terminal clinical events (cardiogenic, neurological, respiratory, or unknown/other).

**Results:**

Two thousand ninety-seven and 1041 children were randomised to bolus vs no bolus, of whom 254 (12%) and 91 (9%) respectively died within 28 days. Median (IQR) bolus fluid in the bolus groups received by 4 h was 20 (20, 40) ml/kg and was the same at 8 h; total fluids received in bolus groups at 4 h and 8 h were 38 (28, 43) ml/kg and 40 (30, 50) ml/kg, respectively. Total fluid volumes received in the control group by 4 h and 8 h were median (IQR) 10 (6, 15) ml/kg and 10 (10, 26) ml/kg, respectively. Mortality risk was greatest 30 min post-randomisation in both groups, declining sharply to 4 h and then more slowly to 28 days. Maximum mortality risk was similar in bolus and no bolus groups; however, the risk declined more slowly in the bolus group, with significantly higher mortality risk compared to the no bolus group from 1.6 to 101 h (4 days) post-randomisation. The delay in decline in mortality risk in the bolus groups was most pronounced for cardiogenic modes of death.

**Conclusions:**

The increased risk from bolus therapy was not due to a mechanism occurring immediately after bolus administration. Excess mortality risk in the bolus group resulted from slower decrease in mortality risk over the ensuing 4 days. Thus, administration of modest bolus volumes appeared to prevent mortality risk declining at the same rate that it would have done without a bolus, rather than harm associated with bolus resulting from a concurrent increased risk of death peri-bolus administration.

**Trial registration:**

ISRCTN69856593. Date of registration 15 December 2008.

## Background

The Fluid Expansion As Supportive Treatment (FEAST) randomised controlled trial in African children with shock found a 3.3% absolute increase in mortality at 48 h and a 3.4% increase by 28 days, in those given boluses of fluid (20–40 ml/kg over 1 h of 5% albumin or 0.9% saline) compared to a control group given maintenance fluid (4 ml/kg/hr 5% dextrose or others following national guidelines) [1]. It has been well documented that child mortality rates shortly following hospital admission in sub-Saharan Africa are high with, for example, 14% of in-hospital deaths occurring within 4 h of admission in a prospective study in Kenya [2]. However, how mortality risk changes over time post-admission in this setting has not been explored. Previous analyses to understand mortality risk in the FEAST trial focussed either on describing the mode of death [3] or on mortality prediction from baseline measures [4]. Currently, the mechanisms by which bolus increased mortality risk remain unclear [3]. Examining how and when mortality risk changed over time in those receiving and not receiving boluses could provide insights into the mechanisms and guide future research, including clinical trials. Different hypotheses could be that the excess mortality risk was restricted to the period around the bolus administration, meaning that the maximum mortality experienced by children receiving boluses was greater than controls. Alternatively, mortality risk could differ by the same amount throughout admission/follow-up, or differences in risk could predominantly be later during admission/follow-up. We therefore used flexible parametric models to characterise changes in mortality risk after randomisation and investigate whether, and for how long, any increased risk from receiving a bolus was maintained.

## Methods

### Trial design and population

The FEAST trial was conducted in six centres (large regional referral hospitals and small district hospitals) across three countries (Kenya, Uganda, and Tanzania) from 2009 to 2011 [1] (ISCRTN 69856593). Briefly, children aged 2 months to 12 years with severe febrile illness (pyrexia (≥ 37.5 °C) or hypothermia (< 36 °C)), one or both of impaired consciousness and respiratory distress, and clinical evidence of impaired perfusion (definitions given in Table 1) were included. Children with severe malnutrition, burns, trauma, gastroenteritis, or a presumed non-infectious cause of severe illness were excluded. Three thousand one hundred forty-one children without severe hypotension were randomised to receive boluses of 20 ml/kg of 5% human albumin solution or 0.9% saline solution over 1 h, or maintenance fluids only at 4 ml/kg/hr (no bolus control group) and are included in this retrospective post hoc analysis. In July 2010, a protocol amendment stipulated that children in the bolus groups who had persisting signs of impaired perfusion at 1 h should have a second 20 ml/kg bolus following their randomised allocation (total 40 ml/kg). Standardised case report forms were completed at enrolment, at specific time points during the first 48 h, and at a 28-day post-discharge follow-up visit. Throughout the hospital admission, severe adverse events were reported immediately and clinical features of suspected pulmonary oedema, raised intracranial pressure, evidence of hypovolaemia/shock, and allergic event were actively solicited.

**Table 1:** Baseline characteristics, interventions, and mortality

	Randomised group			Total (*n* = 3141)
	Albumin bolus (*n* = 1050)	Saline bolus (*n* = 1047)	Control (no bolus) (*n* = 1044)	
Baseline characteristics				
Age (median (IQR) months)	23 (14, 37)	23 (13, 37)	25 (14, 40)	24 (13, 38)
Female sex	474 (45%)	480 (46%)	498 (48%)	1452 (46%)
Malaria^a^	590 (57%)	612 (59%)	593 (57%)	1795 (57%)
Impaired consciousness^b^	811 (77%)	828 (79%)	759 (73%)	2398 (76%)
Respiratory distress	874 (83%)	854 (82%)	857 (83%)	2585 (83%)
Impaired perfusion				
Capillary refill > 2 s	263 (25%)	299 (29%)	257 (25%)	819 (26%)
Lower limb temperature gradient^c^	620 (59%)	629 (61%)	610 (58%)	1859 (59%)
Weak radial pulse	210 (20%)	239 (23%)	206 (19%)	655 (21%)
Severe tachycardia^d^	736 (70%)	721 (69%)	738 (71%)	2195 (70%)
Interventions				
One or more boluses received	1045 (99%)	1041 (99%)	1 (< 1%)	2087 (67%)
Two or more boluses received	318 (30%)	306 (29%)	6 (< 1%)	630 (20%)
Mortality				
Deaths by 48 h	111 (11%)	110 (11%)	76 (7%)	297 (9%)
Deaths by 28 days	128 (12%)	126 (12%)	91 (9%)	345 (11%)
Cardiogenic deaths	56 (5%)	51 (5%)	29 (3%)	137 (4%)
Neurological deaths	33 (3%)	33 (3%)	36 (3%)	102 (3%)
Respiratory deaths	27 (3%)	29 (3%)	16 (2%)	72 (2%)
Unknown/other TCE	12 (1%)	12 (1%)	10 (1%)	34 (1%)
Median time to death (hours) (IQR)^e^	10.4 (2.8, 26.5)	8.1 (2.9, 21.8)	7.8 (1.8, 27.0)	8.5 (2.5, 23.8)

^a^Positive for malaria parasitaemia on either a rapid diagnostic test or a microscopy slide

^b^Impaired consciousness was defined as prostration or coma. Prostration: the inability of a child older than 8 months of age to sit upright or the inability of a child 8 months of age or younger to breast-feed. Coma: the inability to localise a painful stimulus)

^c^Lower limb temperature gradient, defined as a notable temperature change from cold (dorsum of foot) to warm (knee) when running the back of hand from the toe to the knee

^d^Weak pulse: weak radial pulse or severe tachycardia, defined as heart rate > 180 beats per minute (bpm) for children < 1 year old, > 160 bpm for those 1 to 4 years old, and > 140 bpm for those ≥ 5 years old)

^e^Median test *p* = 0.53 comparing the two bolus groups

The Endpoint Review Committee (ERC) had access to ‘blinded’ clinical narratives, bedside vital observations, laboratory baseline, 24-h chemistry, microbiology, malaria and HIV status, and concomitant treatments. During the trial, all deaths were adjudicated by the ERC (blind to randomised group) who assigned terminal clinical events (TCEs) and relationship to fluids following pre-defined definitions. Adjudications were done by a minimum of two members; where there was disagreement, consensus was reached by discussion or additional ERC members included to provide a final adjudication based on the majority decision. TCEs were defined as follows:

1. *Cardiogenic/cardiovascular collapse:* Signs of shock at the point of death—severe tachycardia or bradycardia plus one of prolonged capillary refill time > 2 s, cold peripheries or low SBP (undefined), or where hypoxia was present but circulatory failure was deemed to be the primary problem.
2. *Respiratory:* Ongoing or development of hypoxia (PaO2 < 90%) with chest signs (crepitations or indrawing), or the attending clinician had assigned the primary cause of death as pneumonia and/or possible pulmonary oedema.
3. *Neurological*: Possible raised intracranial pressure (high SBP or relative bradycardia) or severely reduced conscious level (Blantyre Coma Score ≤ 2), focal neurological signs, abnormal pupil response to light, or posturing at the point of death.
4. *Unknown/other:* Death was not witnessed or it was an unknown or other cause of death. Children could have one or more of these terminal clinical events, but the predominant TCE was assigned as cardiogenic for TCEs assigned as both cardiogenic and neurological, and neurological for TCEs assigned as both neurological and respiratory (largely terminal lung aspiration in a comatose child) [3].

### Statistical analysis

Albumin and saline bolus groups were combined for most analyses, as their mortality was very similar [1]. All comparisons between combined bolus and control groups were performed according to intention to treat. All statistical tests were two sided. We used flexible parametric models to estimate a continuously varying all-cause death rate (hazard, or instantaneous risk of mortality) in person-hours (phrs) over time from randomisation to 28 days, using the exact recorded time of death (in hours and minutes) from time of randomisation (in hours and minutes).

Flexible parametric models have the advantage over a Cox model of allowing direct estimation of the underlying instantaneous mortality risk (hazard), which then means that absolute risks and risk differences between groups (here bolus and control) can be estimated continuously over time, rather than just compared using the hazard ratio. These models can incorporate time-dependent effects of factors on the instantaneous mortality risk and thus can allow the effect of bolus to vary over time, and also allow for mortality risk to increase then decrease or vice versa (non-monotonicity). Children were censored at the earliest of their last clinical assessment or 28 days, and we included a time-dependent effect of bolus (see Additional file 1 for details) [5, 6]. We used the same methods to estimate cause-specific death rates. Sensitivity analyses considered the impact of including deaths occurring between screening and randomisation (based on time from admission in the analysis) [7]. As part of the trial design, verbal consent (assent) was used to avoid delays in treatment [8]. However, a small number of deaths (*n* = 11) occurred between screening and randomisation and were therefore not included in main analysis; a sensitivity analysis included these by randomly allocating a death time from 1 to 11 min from admission (one death per minute) as exact times were not recorded for these deaths. Analysis counted time from admission (median (IQR) 15mins (0–25 min) prior to randomisation), and the bolus and control groups were combined since those dying before randomisation were not assigned a group. Simulations from a piecewise exponential model for survival times based on instantaneous mortality risks estimated from the trial data were used to assess variability in estimates of mortality risk shortly after randomisation (20 datasets of 3000 observations) (further detail in Additional file 1).

All analyses were performed using Stata software version 14.2.

## Results

There were 3141 children randomised between albumin bolus, saline bolus, and control in FEAST (2097 and 1041 to the combined bolus and control groups, respectively). Median age was 24 months; 1795 (57%) had malaria (Table 1). By 28 days, there were 345 deaths (11%), and 87 (3%) children were lost to follow-up: 254/2097 deaths (12%) and 65/2097 lost (3%) in the bolus group and 91/1044 deaths (9%) and 22/1044 lost (2%) in the control group [1]. Adherence to fluid administration was high with 2086/2097 (99%) children randomised to the bolus groups receiving a bolus. Median (IQR) bolus fluid in the bolus groups received by 4 h was 20 (20, 40) ml/kg and was the same at 8 h; total fluids received in bolus groups at 4 h and 8 h were 38 (28, 43) ml/kg and 40 (30, 50) ml/kg, respectively. Total fluids received in the control group by 4 h and 8 h were median (IQR) 10 (6, 15) ml/kg and 10 (10, 26) ml/kg, respectively. In the control group, 7 (< 1%) children received a 20-ml/kg saline bolus within 4 h (Table 1) with all children receiving maintenance fluids at 4 ml/kg/hr.

### All-cause mortality risk over time

**Fig. 1:**
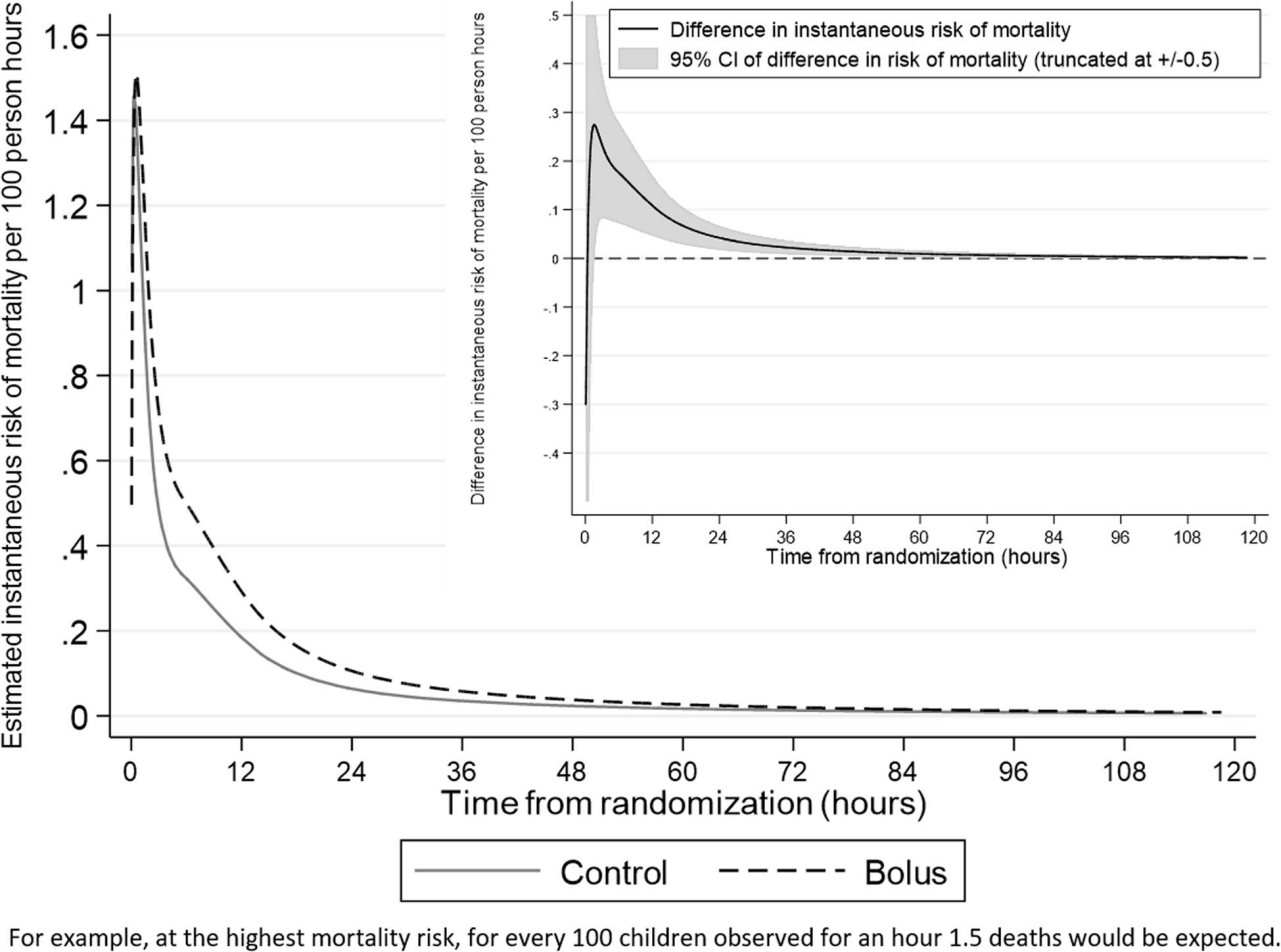
Mortality risk in the first 5 days (120 h) post-randomisation (main graph) and difference (bolus-control) in risk (inset graph)

**Fig. 2:**
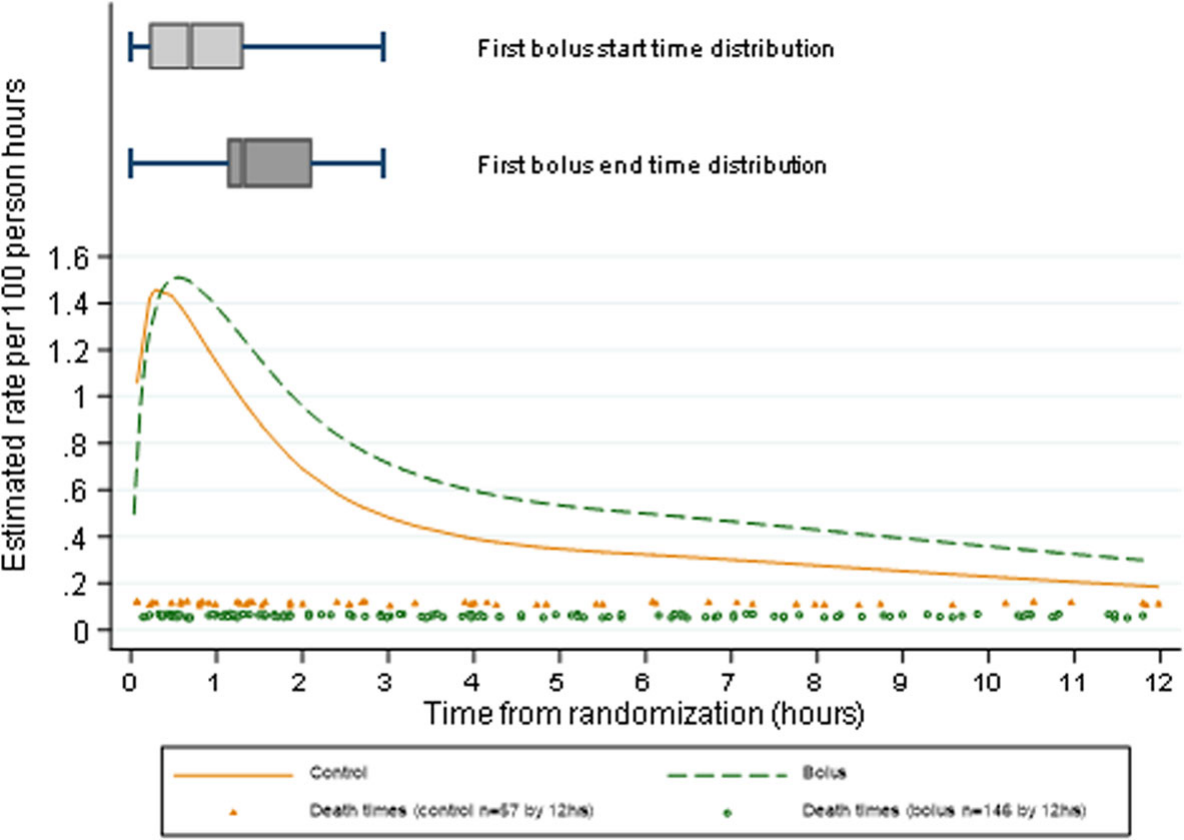
Mortality risk, bolus start and end times, and death times in the first 12 h post-randomisation

From randomisation (0 h), mortality risk was estimated to increase to a maximum around 0.5 h post-randomisation in both groups, then declined sharply to 4 h and continued to decline more slowly from 4 to 16 h (Figs. 1 and 2), and even more slowly through to 120 h (Fig. 1) and to 28 days (Additional file 1: Figure S1). The difference in mortality risk between bolus vs control groups was statistically significant between 1.6 and 101 h (4 days) post-randomisation (lower bound of pointwise 95% CI for the difference > 0), with the greatest difference in the first 12 h (Fig. 1, Additional file 1: Table S1). The maximum mortality risk was however similar in the two groups (Figs. 1 and 2). Mortality risk over time and maximum mortality risk were very similar between the albumin bolus and saline bolus groups (Additional file 1: Figure S2).

The median start time for the first bolus was 41 min (interquartile range (IQR) 13–78) post-randomisation; the first bolus administration ended a median 78 min post-randomisation (IQR 67–126) (Fig. 2). However, mortality risk was highest prior to when the majority of boluses were started (33 min post-randomisation), with a similar maximum observed in the control group mortality risk (at 17 min post-randomisation).

### Timing of maximum mortality risk

The early high mortality risk soon after randomisation is consistent with the high immediate mortality risk of critically ill children at triage and admission and/or some children presenting in a pre-terminal state and dying from severe shock. Supporting this, 26 of the 39 deaths in the first hour from randomisation were from cardiogenic TCEs and were evenly distributed across randomisation groups (Additional file 1: Table S2). However, Fig. 2 suggests that mortality risk rises in both bolus and control groups during the first 30 mins from randomisation, a risk which is not consistent with high immediate mortality risk nor the fact that the actual death times appeared fairly evenly distributed across the first 2 h post-randomisation (Fig. 2). We therefore considered whether the early rises in mortality risk from randomisation to ~ 30 min could be due to trial design or analysis method in two sensitivity analyses.

One possibility is that estimated mortality risk in trial data rises because moribund patients are not consented (even though the initial consent was verbal in the trial). The sensitivity analysis including deaths between screening and randomisation (see the ‘Methods’ section for details) showed that including these deaths only slightly increased the estimated mortality risk around admission, and mortality risk was still estimated to rise to a similar maximum level around 20 min from admission (Additional file 1: Figure S3). However, the 95% confidence intervals for the estimated mortality risk were wide and consistent with high but non-varying mortality risk in the first 1–2 h post-randomisation.

A second sensitivity analysis investigated the potential for the analysis method to artificially induce an apparent rise in risk in the first hour post-randomisation using simulations. This suggested that initial estimated increases in risk were most likely to have been created by the statistical modelling process attempting to accurately describe the timing of specific early deaths post-randomisation when by definition all deaths have to occur after time zero (details in Additional file 1).

Therefore, overall results were consistent with mortality risk being high, but not varying, during the first 1–2 h post-randomisation, before declining in both groups, but more slowly in those who had received a bolus.

### Cause-specific mortality risk over time

**Fig. 3:**
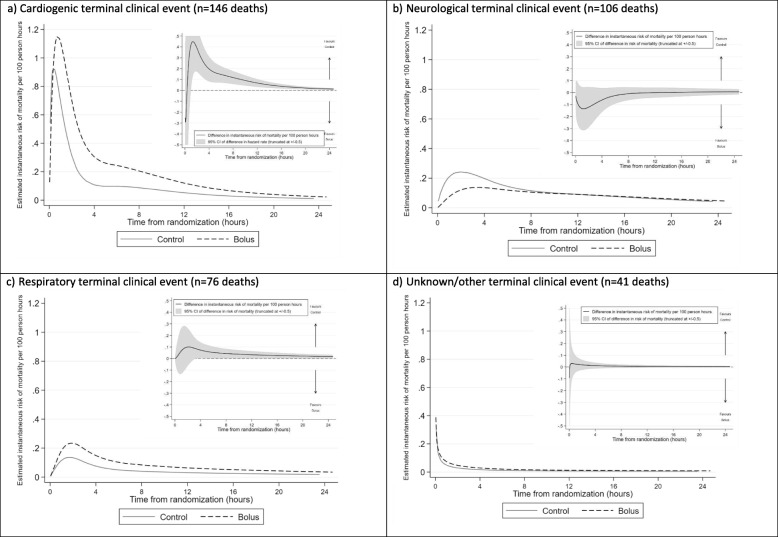
Cause-specific mortality risk in the first 24 h post randomisation (main graphs) and difference (bolus-control) in risk (inset graphs). **a** Cardiogenic terminal clinical event (*n* = 146 deaths). **b** Neurological terminal clinical event (*n* = 106 deaths). **c** Respiratory terminal clinical event (*n* = 76 deaths). **d** Unknown/other terminal clinical event (*n* = 41 deaths)

The most common cause of death was a cardiogenic TCE (Table 1, Fig. 3). Although the maximum cardiogenic mortality risk was slightly higher in the bolus group than in the control group (1.2 vs 0.9 deaths per 100 phrs), again, the main impact of bolus was to extend the period of higher risk vs control, statistically significant until 33 h post-randomisation (lower bound of 95% CI of the difference > 0; there is lower power to detect differences in cause-specific deaths compared to all deaths over longer durations). There was no statistical evidence of differences between bolus and control groups for risk of other causes of death over time, although point estimates suggested that excess risk of death from a respiratory TCE persisted for longer post-randomisation in the bolus groups, similar to risk of death from a cardiogenic TCE.

## Discussion

The FEAST trial showed a 3.3% absolute increase in risk of mortality by 48 h (and a 3.4% increase by 28 days) for those in the bolus groups compared to the control group [1]. The amount of fluid given as a bolus and rate was modest, in contrast to current recommendations, and the majority of children received only one 20 ml/kg bolus given over 1 h. Here, we investigated temporal changes in absolute increased mortality risk post-admission. Whilst it could have been hypothesised that bolus administration directly increased immediate mortality risk compared to controls around the time that the bolus was given, in fact, we found that mortality risk in children in the bolus groups simply declined more slowly than in the control group (Fig. 2). Overall mortality risk was at its maximum at around 30 min post-randomisation in both groups, consistent with children been admitted when acutely sick and at high risk of mortality, but we found no evidence that the excess mortality risk from bolus observed overall in the trial was concentrated in the time period immediately after bolus administration, nor that the maximum mortality risk differed between bolus and control. However, the detrimental impact of receiving a bolus continued beyond this acute phase up to 4 days after randomisation. Thus, the main effect of boluses was to delay ‘normalisation of risk of mortality’ following hospital admission, such that mortality risk in children receiving boluses declined at a slower rate compared with those not receiving boluses. This was a consistent finding with all statistical approaches. This is also in agreement with previous reported additional analyses, and active clinical monitoring of solicited serious adverse event by sites and the blinded ERC indicating cardiovascular collapse was the main TCE which contributed most substantially to the excess mortality in the bolus groups compared to control rather than increasing the fluid overload events (i.e. raised intracranial pressure or respiratory TCE) [3].

The WHO continue to advise that for children with three features of impaired perfusion (WHO definition of shock: all 3 of (i) cold extremities AND (ii) capillary refill time MORE than 3 s AND (iii) weak and a fast pulse (definition of tachycardia not stated)) 10–20 ml/kg over 30–60 min, and a further 10 ml/kg over 30 min repeating two times for uncorrected shock, should be given [9]. In the FEAST trial, which assent/consent processes permitted enrolment of very sick children, only a very small number had WHO shock (*n* = 65). In this sub-group, 48% of bolus recipients died compared to 20% of non-bolus control group with an increased absolute risk of 28% and relative risk increase of 240% (*p* = 0.07 (two-sided Fisher’s exact test)) [10]. A recent appraisal of published and unpublished literature to determine the frequency of WHO shock in unselected paediatric cohorts admitted to hospital showed that WHO shock criteria were present in very few cases (101/91,952 (~ 0.1%)) and overall these children have a very high mortality (up to 100%) [11]. In the light of these recommendations, the findings of the current analysis, demonstrating that a conservative fluid bolus volume given over a longer period of time increased the risk of mortality for up to 4 days post-bolus, is of concern.

There has been intense speculation around the mechanisms underlying the excess mortality from fluid-bolus therapy in the FEAST trial [12]. One hypothesis we suggested was a ‘re-perfusion’ injury where fluid resuscitation acts a ‘microcirculatory flush’ mechanism, mobilising products of catabolism, pro-inflammatory peptides, and toxins, and resulting in multiple downstream effects including myocardial ‘stunning’, organ damage, and poor subsequent outcomes [3, 13]. Our analyses support this hypothesis by showing that mortality risk in the bolus group declined more slowly; the hypoperfused state, involving both macro- and microvascular systems, may thus be serving as a protective physiological response, preventing a vascular systemic spread of pathogens or toxins, which subsequently occurred with re-perfusion of ‘global’ ischaemia [14, 15].

This would also support our previous findings that the adverse effects of bolus fluid resuscitation were mediated via their impact on vascular haemodynamics and myocardial performance [1, 3]. In an ovine endotoxin model of sepsis, which investigated a two-hit model (endotoxin and saline bolus therapy), we reported marked increases in cardiac troponin, a marker of myocyte death, at 16 h in the sepsis group resuscitated with saline [16]. In addition, Atrial Natriuretic Protein (a marker of atrial ‘stretch’) rose significantly in the fluid-resuscitated group, and median ANP was significantly higher than in the no resuscitation group (*p* = 0.02) over the cross-sectional time series. This was concurrent with an increased vasopressor requirement in the fluid-resuscitated group and increased levels of glycocalyx glycosaminoglycan hyaluronan (a marker of glycocalyx damage of the microcirculation). This aligns with our observation that the largest difference in mortality risk in FEAST was seen in those that had a cardiogenic terminal clinical event (TCE) (i.e. cardiovascular collapse) and that decline in mortality risk from cardiogenic deaths was most delayed in the bolus group [3]. In a multi-centre open-label phase II randomised controlled trial evaluating slower versus rapid rehydration strategies in 122 Ugandan/Kenyan children with severe dehydration secondary to gastroenteritis, we also noted that ANP levels rose substantially by 8 h in both arms and the abnormally high levels of ANP persisted to day 7 [17]. Troponin I (a marker of cardiac muscle injury independent of volume status) also rose following fluid administration at 8 h and 24 h in both groups; however, values remained in normal ranges. Both these studies indicate that volume loading, irrespective of rate, may have adverse consequences on the cardiovascular physiology in severe illness. As this was not anticipated during the FEAST trial, we have not been able to explore biomarkers of cardiac damage post-fluid bolus from the FEAST biobank, as we did not store samples post-bolus administration. Risks of death from neurological and respiratory TCEs were much lower and declined slowly in both groups, whereas deaths from unknown/other TCE predominated very shortly after randomisation (and before completion of fluid bolus administration), thus being unlikely to be due to the detrimental effect of fluid overload.

The maximum mortality risk was similar in bolus and control groups, and occurred very close to randomisation, prior to completion of the majority of boluses. The screening and consent process in clinical trials usually prevents randomisation of children in a pre-terminal state, and this has been hypothesised to cause estimates of increasing risk shortly after randomisation when these type of models have been used in other applications, such as advanced HIV disease [18]. However, FEAST had an emergency assent process, so that critically ill children could be randomised quickly, and full consent obtained when the child had stabilised. Further, our sensitivity analyses suggested that the very early increases in estimated mortality risk prior to the maximum risk were likely to have been due to the statistical modelling process, rather than a clinical increase in risk during the first hour. The estimated mortality risk from the model fitted to the data is also consistent with an underlying high constant mortality risk across the first hour, as suggested by the simulated data, which is clinically feasible.

In the FEAST trial, standard supportive treatments included oxygen therapy, rapid correction of hypoglycaemia, and anti-pyretics; to comply with surviving sepsis recommendations, all children received immediate treatment with parenteral antibiotics (i.e. within the first hour) [19]. The high early mortality raises concern as to whether other aspects of early supportive care may have adverse consequences. Although discussed in the literature [20], prospective evaluation will be challenging as much of early supportive care is an intrinsic part of clinical practice. Literature on toxin release by bactericidal antibiotics, including the Jarisch-Herxheimer reaction and pyrexia control, remains both controversial and debatable topics. Few trials have been prospectively designed to address the possible adverse consequences of early supportive care in the critically sick patient [21].

The main study strength is its novel application of flexible parametric survival models, enabling us to further elucidate potential mechanisms. To our knowledge, no studies have applied such models to critically ill children in resource-limited settings, and our application highlights their potential. By necessity, however, one limitation is that it is a post hoc retrospective analysis of prospectively collected trial data. Other study limitations include the lack of detailed data on causes of death and the absence of longitudinal stored samples for the exploration of explanatory hypotheses. Further, there may be some minor inaccuracies in time of death (recorded to the nearest minute); however, all children were managed by a dedicated trial team, a dedicated trial clock was provided to ensure accuracy of timing of observations, and both randomised groups were monitored closely, for example, for signs of fluid overload or need for other supportive treatments.

## Conclusions

This more detailed analysis of the FEAST trial data demonstrates that fluid boluses did not substantially increase mortality risk around the time of administration, but rather delayed the reduction of mortality risk for at least 4 days, preventing mortality risk declining at the same rate as it would without a bolus.

## Supplementary information

**Additional file 1.** Description of flexible parametric models and description of simulations to investigate analysis methods. Table S1. Difference in mortality risk (bolus-control) over time from randomisation. Table S2. Causes of death in 39 children dying within the first hour of randomisation. Figure S1. All-cause mortality risk over 28 days of follow up (main graph) and over the first 48 hours (inset graph) from randomization (all randomised groups combined). Figure S2. Mortality risk and death times in the first 12 hours post-randomisation by bolus fluid type compared to no bolus. Figure S3. All-cause mortality risk including children dying before randomisation. Figure S4. Mortality risk over the first 12 hours from 20 simulated datasets (modelling hazard over time).

## Data Availability

The datasets analysed during the current study are available from the corresponding author on reasonable request.
