# Supplementary material for: Mortality risk over time after early fluid resuscitation in African children
Source: Crit Care. 2019 Nov 27;23:377. doi: 10.1186/s13054-019-2619-y (PMC6882199; doi:10.1186/s13054-019-2619-y)
Supplement: Supplementary file 1 — Additional file 1. Description of flexible parametric models and description of simulations to investigate analysis methods. Table S1. Difference in mortality risk (bolus-control) over time from randomisation. Table S2. Causes of death in 39 children dying within the first hour of randomisation. Figure S1. All-cause mortality risk over 28 days of follow up (main graph) and over the first 48 hours (inset graph) from randomization (all randomised groups combined). Figure S2. Mortality risk and death times in the first 12 hours post-randomisation by bolus fluid type compared to no bolus. Figure S3. All-cause mortality risk including children dying before randomisation. Figure S4. Mortality risk over the first 12 hours from 20 simulated datasets (modelling hazard over time). [file 13054_2019_2619_MOESM1_ESM.docx]

Supplementary Appendix:

Methods

Description of flexible parametric models:

Flexible parametric models (FPMs) (also called Royston Parmar models) (1) provide smooth estimates of the hazard and survival function for any combination of covariate values and can model non-monotonic mortality risks on a proportional odds, proportional hazard or probit scale. They use a restricted cubic spline function of log time, with the cubic polynomial segments smoothly joined at values (knots). To identify both the best fitting scale as well as the most appropriate number of interior knots, the Akaike information criterion (AIC) of the three unadjusted FPMs on different scales with no covariates with a range of 0 to 5 knots (1 to 6 degrees of freedom) were compared (2). The best fitting models according to AIC on each scale were within 3.84 (χ^2^(1)) of each another. As previous work looking at survival in FEAST trial data has used the (proportional hazards) Cox model and as the fit of the proportional hazards models and proportional odds models were comparable, the proportional hazards scale was chosen for ease of comparison and interpretation of hazard ratios as estimates. A time-dependent effect of bolus was then included by forming interactions with the spline terms allowing the time-dependent effect to have a different number of knots and at different locations than the baseline effect. AICs of FPMs on a proportional hazard scale with 0 to 3 knots for the time-dependent effect of bolus and 0 to 5 knots for the underlying time effect were compared. The best fitting model on a proportional hazard scale had 4 interior knots (at the 20^th^, 40^th^, 60^th^, 80^th^ percentiles of the uncensored survival times) plus 2 boundary knots at the minimum and maximum of uncensored survival times for the underlying time function, and 1 knot at the 50^th^ percentile plus two boundary knots at the minimum and maximum for the time dependent effect of bolus. This model was also used for sensitivity analyses and to fit cause-specific hazards for death from the predominant terminal clinical event (TCE) (censoring deaths from other TCE) (3).

Description of simulations to investigate analysis methods

To investigate the potential for the analysis method to artificially induce an apparent rise in risk in the first hour, a piecewise exponential model was fitted to the trial data with changepoints at 1, 2, 3, 6, 12, 24 and 48 hours from randomization and the interval-specific rates estimated. These were then used to simulate 20 datasets with 3000 observations and survival times following this piecewise exponential distribution with genuine constant high risk in the first hour post randomization. Flexible parametric models for the log cumulative hazard scale were then fitted to the simulated data. The predicted hazard from these parametric models, with the same number of knots as found for the flexible parametric model fitted to the log cumulative hazard in the trial data (5 df, 4 internal knots, as above), was estimated and plotted to explore how the models dealt with estimates of constant high hazard immediately after randomization (when time = 0) which then dropped at each hour from randomization. All 20 simulations estimated fitted on the log cumulative hazard scale estimated that early mortality risk initially increased immediately after randomisation, albeit by varying magnitudes, before reaching a maximum (Supplementary Appendix Figure 4).

Supplementary Table 1: Difference in mortality risk (bolus-control) over time from randomisation

| Time from randomization (hours) | Difference in mortality risk per 100 person-hours (95% CI ) |
| --- | --- |
| 1 | 0.24 (-0.19, 0.68) |
| 2 | 0.26 (0.05, 0.48) |
| 6 | 0.18 (0.07, 0.27) |
| 12 | 0.11 (0.05, 0.17) |
| 24 | 0.04 (0.02, 0.06) |
| 48 | 0.01 (0.005, 0.02) |
| 72 | 0.007 (0.001, 0.012) |
| 96 | 0.003 (0.0001, 0.007) |
| 120 | 0.003 (-0.0002, 0.007) |

Supplementary Table 2: Causes of death in 39 children dying within the first hour of randomisation

| Terminal Clinical Event | Albumin Bolus | Saline Bolus | Control | Total |
| --- | --- | --- | --- | --- |
| Cardio respiratory | 9 | 8 | 9 | 26 |
| Neurological | 1 | 1 | 1 | 3 |
| Respiratory | 0 | 2 | 1 | 3 |
| Unknown/Other | 3 | 1 | 3 | 7 |
| Total | 13 | 12 | 13 | 39 |

Supplementary Figure 1: All-cause mortality risk over 28 days of follow up (main graph) and over the first 48 hours (inset graph) from randomization (all randomised groups combined).

Supplementary Figure 2: Mortality risk and death times in the first 12 hours post-randomisation by bolus fluid type compared to no bolus.

Supplementary Figure 3: All-cause mortality risk including children dying before randomisation

Supplementary Figure 4: Mortality risk over the first 12 hours from 20 simulated datasets (modelling hazard over time)

References

1. Royston P, Parmar MK. Flexible parametric proportional-hazards and proportional-odds models for censored survival data, with application to prognostic modelling and estimation of treatment effects. Stat Med. 2002;21(15):2175-97.

2. Lambert PC, Royston P. Further development of flexible parametric models for survival analysis. Stata J. 2009;9(2):265-90.

3. Lambert PC, Wilkes SR, Crowther MJ. Flexible parametric modelling of the cause-specific cumulative incidence function. Stat Med. 2016.
